# Sevoflurane impairs learning and memory of the developing brain through post-transcriptional inhibition of CCNA2 via microRNA-19-3p

**DOI:** 10.18632/aging.101673

**Published:** 2018-12-12

**Authors:** Xin Zhao, Yanwu Jin, Haibo Li, Yuxiu Jia, Yuelan Wang

**Affiliations:** 1 Department of Anesthesiology, the Second Hospital of Shandong University, Jinan 250033, China; 2 Operating Room, Jinan Central Hospital, Affiliated to Shandong University, Jinan 250013, China; 3 Department of Research, the Second Hospital of Shandong University, Jinan 250033, China; 4 Department of Anesthesiology, Qianfoshan Hospital of Shandong Province, Affiliated to Shandong University, Jinan 250014, China

**Keywords:** sevoflurane (SEVO), miR-19-3p, CCNA2, developing brain

## Abstract

The molecular mechanisms underlying sevoflurane (SEVO)-induced impairment of learning and memory remain unclear. Specifically, a role of microRNAs (miRNAs) in the control of the neuron proliferation in the developing brain exposed to SEVO has not been reported previously. Here, we studied the effects of SEVO exposure on the neural cell proliferation, and on the learning and memory of neonatal rats. We found that SEVO exposure significantly decreased neuron cell proliferation, reduced BDNF levels in brain, and impaired learning and memory of neonatal rats in Morris water maze test and Plus-Maze discriminative avoidance task (PM-DAT), likely through downregulation of CCNA2 protein. Next, we used bioinformatic tools to predict CCNA2-binding microRNAs (miRNAs), and found that miR-19-3p was upregulated in neurons exposed to SEVO. Moreover, miR-19-3p functionally inhibited the protein translation of CCNA2 in a human neural cell line, HCN-2. Furthermore, intracranial injection of adeno-associated virus carrying antisense of miR-19-3p under a CMV promoter into the neonatal rats significantly alleviated SEVO exposure-induced impairment of neuron cell proliferation, as well as the learning and memory of the rats. Together, our data suggest that SEVO-induced upregulation of miR-19-3p post-transcriptionally inhibits CCNA2, which contributes to the SEVO-associated impairment of learning and memory of the neonatal rats.

## Introduction

Whether sevoflurane (SEVO) may affect developing brain and cause learning and memory impairment is still under debate. Some studies have shown that SEVO are toxic to neuron cells in the developing brain, resulting in long-term deficits in neurocognition and learning ability [1–7], while others have disagreed with at least parts of these conclusions [8–11]. The discrepancy largely results from the insufficient understanding of the molecular mechanisms underlying the experimental observed phenotype.

The detrimental effects of SEVO have been proposed in some studies. For example, SEVO induced activation of gamma-aminobutyric acid (GABA) and suppression of N-methyl-D-aspartate (NMDA) receptors [12–14]. GABA is the most important inhibitory neurotransmitter in the central nervous system, and excessive excitability of GABA receptors by SEVO caused inflow of extracellular calcium ions, neuronal calcium overload and neurotoxicity, resulting in long-term cognitive impairment [12–14]. Moreover, blocking NMDA receptors by SEVO can reduce extracellular glutamate concentration, synapse formation and intercellular neuron connections [15]. When the developing brain is exposed to SEVO, it further activated synaptic NMDA receptors, which mediated the increase of calcium influx with oxygen free radicals to induce neuronal apoptosis in the hippocampus that impairs learning and memory functions [16]. Brain-derived neurotrophic Factor (BDNF) is an extremely important neurotrophic factor that promotes synaptic plasticity. Exposure to SEVO has been shown to reduce BDNF levels, and thus affect the spatial memory ability of rats in a serine/threonine acid kinase (Akt)/glycogen synthase kinase 3β (GSK-3β) signaling-dependent manner [17]. However, the effects of SEVO on the growth of neurons in the developing brain are poorly understood. Especially, a role of microRNAs (miRNAs) in the SEVO-mediated impairment of neuron growth and function has not been reported previously.

Proliferation of eukaryotes in a complete cell cycle comprises of G0 phase, G1 phase, S phase, G2 phase and M phase [18]. The most important cell-cycle transitions of an active cell cycle are G1/S phase transition and G2/M phase transition, which are tightly regulated by different cyclin-dependent kinases (CDKs) and their activating cyclin subunits [18]. Members of the CDK and cyclin families build up interchangeable binding surfaces formed by different cyclin-CDK combinations. Cyclin E (CCNE)–CDK2 is important for mediating S phase initiation [18]. Cyclin A (CCNA), binding to either CDK1 or CDK2, is important for maintaining S phase progress and mitotic initiation [18]. Cyclin B (CCNB), binding with CDK1, introduces mitosis entry [18]. So far, the effects of SEVO on these CDKs and cyclins on the neurons of developing brain are not well established.

Here, we studied the effects of SEVO exposure on the neuron cell proliferation, and on the learning and memory of neonatal rats. We found that SEVO exposure significantly decreased neuron cell proliferation, reduced BDNF levels in brain, and impaired learning and memory of neonatal rats in Morris water maze test and Plus-Maze discriminative avoidance task (PM-DAT), likely through downregulation of protein levels of CCNA2. Since CCNA2 mRNA levels were not altered in SEVO-exposed neurons, which implied presence of post-transcriptional control of CCNA2, we used bioinformatic tools to predict CCNA2-binding miRNAs, and found that miR-19-3p was upregulated in neurons exposed to SEVO. Moreover, miR-19-3p was a CCNA2-targeting miRNA that functionally inhibited the protein translation of CCNA2 in a human neural cell line, HCN-2. Furthermore, intracranial injection of adeno-associated virus that carries antisense of miR-19-3p under a CMV promoter into the neonatal rats significantly alleviated SEVO exposure-induced impairment of neuron cell proliferation, and learning and memory of the rats.

## RESULTS

### SEVO impairs learning, memory and neuron proliferation of neonatal rats

**Figure 1:**
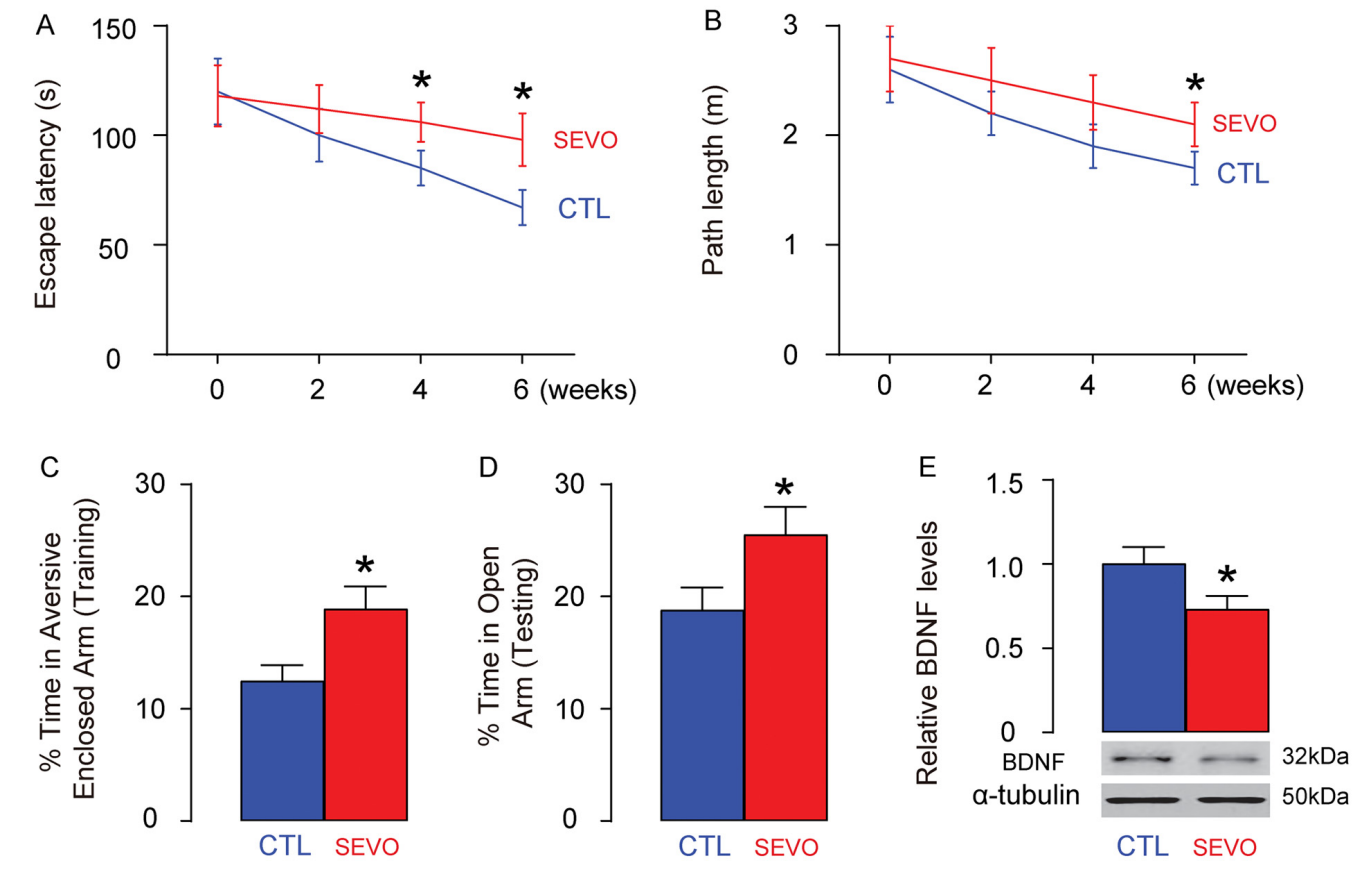
**SEVO impairs learning, memory and neuron proliferation of neonatal rats.** Neonatal rats were separated into 2 groups of ten of each. The control group (CTL) was exposed to normal gas and the SEVO group was exposed to 5% SEVO for 4 hours. (**A**-**B**) Morris water maze test. (**A**) Escape latency. (**B**) Path length. (**C**-**D**) PM-DAT at 6 weeks after SEVO exposure. (**C**) Time spent in aversive arm at training period. (**D**) Time spent in open arm at testing period. (**E**) Western blotting for BDNF in rat brain. (**F**-**G**) Ki-67 staining in the hippocampal area of rat brain, 6 weeks after SEVO exposure, by representative images (**F**), and by quantification (**G**). *p<0.05. N=10. Scale bars are 100µm.

Neonatal rats were separated into 2 groups of ten of each. The control group (CTL) was exposed to normal gas and the SEVO group was exposed to 5% SEVO for 4 hours. Morris water maze test showed that post SEVO exposure, neonatal rats exhibited poorer performance in both escape latency (Figure 1A) and path length (Figure 1B), compared to CTL. Moreover, the rats were also assessed by PM-DAT at 6 weeks after SEVO exposure. We found that post SEVO exposure, neonatal rats exhibited poorer performance in time spent in aversive arm at training period (Figure 1C), and in time spent in open arm at testing period (Figure 1D), compared to CTL. These results suggest that both learning and memory of the neonatal rats are impaired by SEVO exposure. BDNF is an extremely important neurotrophic factor that regulate the survival of existing neurons, and promote the growth and differentiation of new neurons and synapses. BDNF levels were examined in the rat brain 6 weeks after SEVO exposure, showing a significant decrease (Figure 1E). Ki-67 is a marker for proliferating cells, and thus the staining for Ki-67 was performed, showing decreases in Ki-67+ proliferating neuron cells the hippocampal area of rat brain, 6 weeks after SEVO exposure, by representative images (Figure 1F), and by quantification (Figure 1G). Together, these data suggest that SEVO impairs learning, memory and neuron proliferation of neonatal rats.

### SEVO impairs neuron cell proliferation in vitro likely through decreasing CCNA2

**Figure 2:**
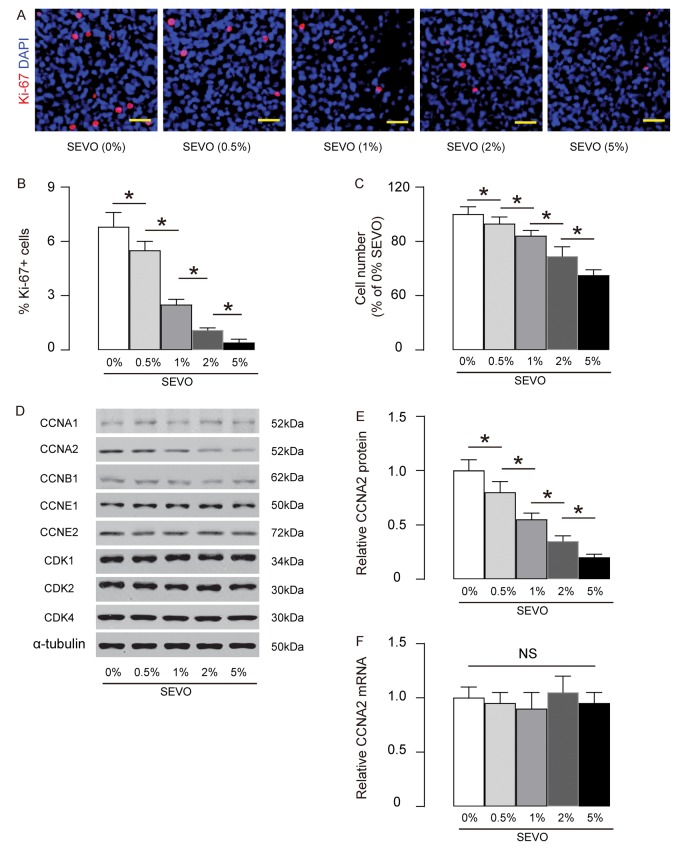
**SEVO impairs neuron cell proliferation in vitro likely through decreasing CCNA2.** Identical number of HCV-2 neural cells was exposed to different doses of SEVO for 48 hours and then subjected to analysis. (**A**-**B**) Ki-67+ staining on HCN-2 cells, shown by representative images (**A**), and by quantification (**B**). (**C**) The total cell number based on DAPI staining. (**D**) Western blotting for CDKs and cyclins in 5% SEVO-exposed HCN-2 cells vs gas-exposed HCN-2 cells. (**E**) Quantification of changes in CCNA2 protein. (**F**) RT-qPCR for CCNA2 mRNA. *p<0.05. NS: non-significant. N=5. Scale bars are 50µm.

Next, we aimed to figure out the mechanisms underlying decreased neuron proliferation by SEVO. Identical number of HCV-2 neural cells was exposed to different doses of SEVO for 48 hours and then subjected to analysis. We found that SEVO dose-dependently decreased the Ki-67+ cells, shown by representative images (Figure 2A), and by quantification (Figure 2B). The total cell number was counted based on DAPI staining, showing that SEVO dose-dependently reduced the total cell number (Figure 2C). Thus, SEVO impairs neuron cell proliferation in vitro. Cell cycle was controlled by different CDKs and cyclins. We examined the levels of all CDKs and cyclins that were expressed in neural cells. We found that only CCNA2 was suppressed by SEVO dose-dependently (Figure 2D-E), but CCNA2 mRNA was not altered in SEVO-exposed HCN-2 cells (Figure 2F). These data suggest that SEVO impairs neuron cell proliferation in vitro likely through post-transcriptional suppression of CCNA2.

### SEVO upregulates miR-19-3p in neuron cells

**Figure 3:**
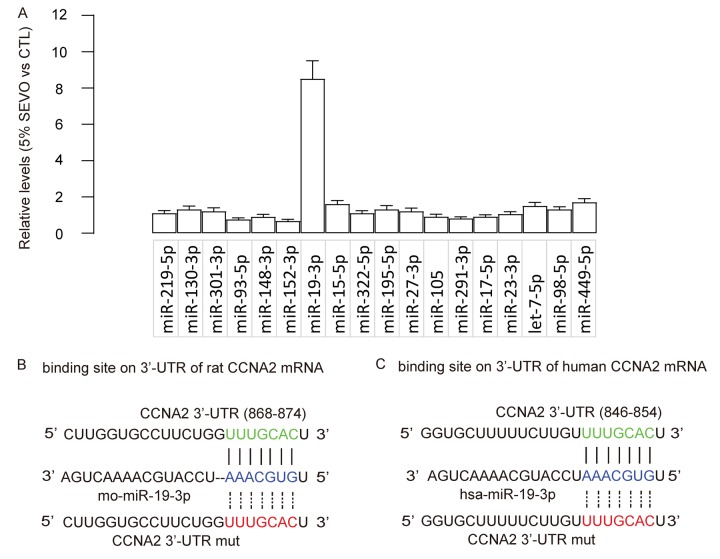
**SEVO upregulates miR-19-3p in neuron cells.** (**A**) RT-qPCR for 18 CCNA2-targeting miRNAs in HCN-2 cells exposed to either 5% SEVO or CTL gas. (**B**-**C**) Bioinformatics analysis showed predictive binding of miR-19-3p onto 3’-UTR of CCNA2 mRNA of rat (**B**) and human (**C**). N=5.

Based on abovementioned data, we hypothesized that SEVO may suppress CCNA2 by activating a CCNA2-targeting miRNA. Bioinformatics analysis showed that rat CCNA2 had 18 targeting miRNAs. The levels of these miRNAs in HCN-2 cells exposed to control gas or 5% SEVO were compared, and we screened for the upregulated targeting miRNAs which should be responsible for the suppression of CCNA2 by SEVO. We found that miR-19-3p was such a distinguished one that was significantly upregulated by SEVO (Figure 3A). Moreover, predictive binding of miR-19-3p onto 3’-UTR of CCNA2 mRNA was conserved on both rat (Figure 3B) and human (Figure 3C).

### MiR-19-3p targets CCNA2 to suppress its translation in neural cells

**Figure 4:**
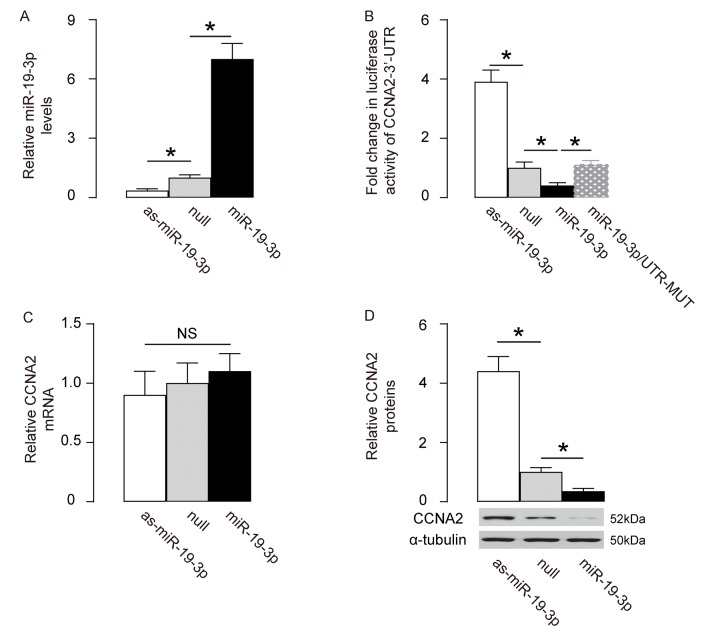
**MiR-19-3p targets CCNA2 to suppress its translation in neural cells.** (**A**) MiR-19-3p was overexpressed by a plasmid carrying miR-19-3p or knocked down by a plasmid carrying as-miR-19-3p in a human neural cell line, HCN-2. Transfection with a null sequence was used as a control. RT-qPCR for miR-19-3p levels in these cells. (**B**) An intact 3'-UTR of CCNA2 mRNA (CCNA2 3'-UTR) and an 3'-UTR of CCNA2 mRNA with a mutant at miR-19-3p-binding site (CCNA2 3'-UTR mut) were prepared. A dual luciferase reporter assay was performed using combinations of one miR-19-3p-modifying plasmid and one CCNA2 3’-UTR plasmid. (**C**-**D**) RT-qPCR (**C**) and Western blot (**D**) for CCNA2 levels in miR-19-3p-modified HCN-2 cells. *p<0.05. NS: non-significant. N=5.

We assessed if the binding of miR-19-3p to CCNA2 is functional. First, miR-19-3p was overexpressed by a plasmid carrying miR-19-3p or knocked down by a plasmid carrying as-miR-19-3p in HCN-2 cells. Transfection with a null sequence was used as a control. The RT-qPCR for miR-19-3p levels was done to confirm the changes in miR-19-3p levels in these cells (Figure 4A). Next, an intact 3'-UTR of CCNA2 mRNA (CCNA2 3'-UTR) and an 3'-UTR of CCNA2 mRNA with a mutant at miR-19-3p-binding site (CCNA2 3'-UTR mut) were prepared. A dual luciferase reporter assay was performed using combinations of one miR-19-3p-modifying plasmid and one CCNA2 3’-UTR plasmid, showing that the specific binding of miR-19-3p to 3’-UTR of CCNA2 mRNA is functional in HCN-2 cells (Figure 4B). In addition, the mRNA levels of CCNA2 was not altered by modification of miR-19-3p levels in HCN-2 cells (Figure 4C), but the protein levels of CCNA2 significantly decreased by overexpressing miR-19-3p, and significantly increased by knocking down miR-19-3p (Figure 4D). Together, our data suggest that MiR-19-3p targets CCNA2 to suppress its translation in neural cells.

### Generation of AAVs that express as-miR-19-3p

**Figure 5:**
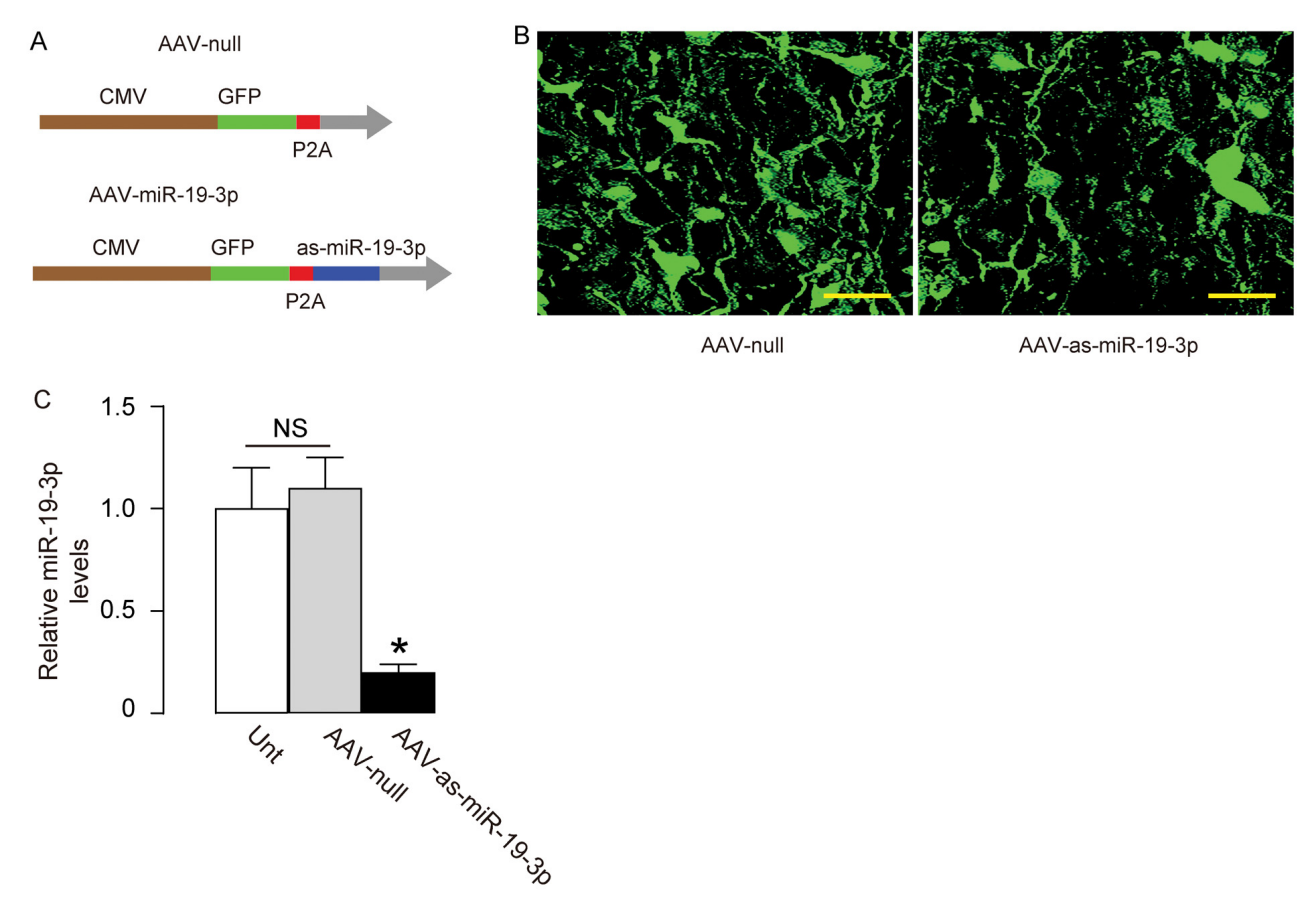
**Generation of AAVs that express as-miR-19-3p.** (**A**) Schematic showing AAVs carrying as-miR-19-3p under the control of a CMV promoter and AAV carrying null under the control of a CMV promoter as a control. The viral backbone had a GFP reporter, which was co-expressed with the transgene, which allowed the transduced cells to be visualized by green fluorescence. (**B**) Transduced HCN-2 cell in culture. (**C**) RT-qPCR for miR-19-3p in AAV-as-miR-19-3p-transduced cells, un-transduced cells (UnT) and null-transduced cells. *p<0.05. NS: non-significant. N=5. Scale bars are 20µm.

In order to assess the effects of suppression of miR-19-3p in brain on the SEVO-induced learning and memory deficit in rats, we prepared AAVs carrying antisense of miR-19-3p (as-miR-19-3p) under the control of a CMV promoter. The control AAV carried null (Figure 5A). The viral backbone had a GFP reporter co-expressed with the transgene, which allowed the transduced cells to be visualized by green fluorescence (Figure 5B). We found that the AAV-as-miR-19-3p reduced about 80% of miR-19-3p levels in transduced HCN-2 cells, compared to un-transduced (UnT) or null-transduced controls (Figure 5C). Thus, these viruses were readily used for in vivo study.

### Abolishment of miR-19-3p upregulation protects neuron proliferation, and learning and memory of rats that are exposed to SEVO

**Figure 6:**
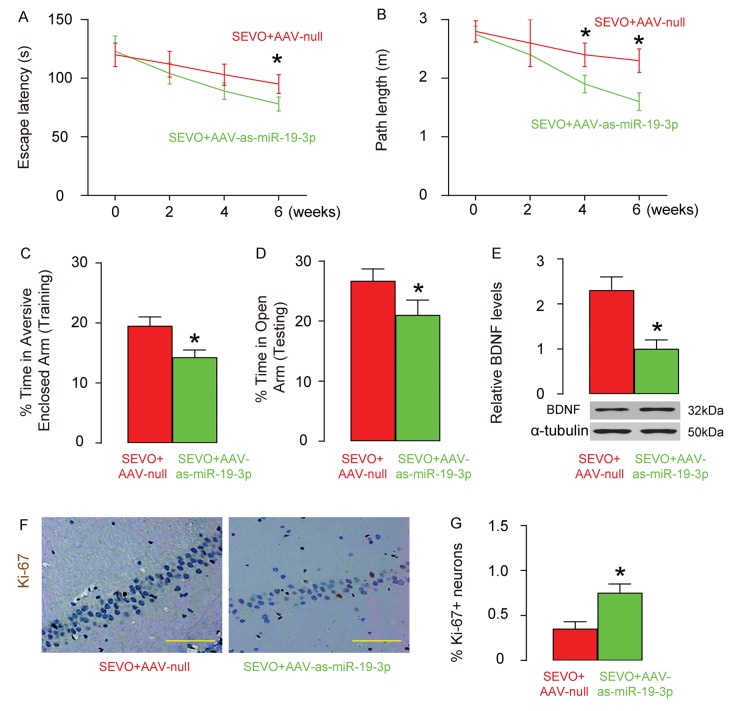
**Abolishment of miR-19-3p upregulation protects neuron proliferation, and learning and memory of rats that were exposed to SEVO.** AAV-as-miR-19-3p or AAV-null was intracranially injected into the bilateral hippocampi of the neonatal rats (n=10 in each group). One weeks after injection, SEVO exposure was performed on these rats. (**A**-**B**) Morris water maze test. (**A**) Escape latency. (**B**) Path length. (**C**-**D**) PM-DAT at 6 weeks after SEVO exposure. (**C**) Time spent in aversive arm at training period. (**D**) Time spent in open arm at testing period. (**E**) Western blotting for BDNF in rat brain. (**F**-**G**) Ki-67 staining in the hippocampal area of rat brain, 6 weeks after SEVO exposure, by representative images (**F**), and by quantification (**G**). *p<0.05. N=10. Scale bars are 100µm.

Finally, we evaluated the effects of abolishment of miR-19-3p upregulation on neuron proliferation, and learning and memory of rats that were exposed to SEVO. AAV-as-miR-19-3p or AAV-null was intracranially injected into the bilateral hippocampi of the neonatal rats (n=10 in each group). One week after injection, SEVO exposure was performed on these rats. Morris water maze test showed that post SEVO exposure, neonatal rats that had received AAV-as-miR-19-3p exhibited significantly better performance in both escape latency (Figure 6A) and path length (Figure 6B), compared to rats that had received AAV-null. Moreover, the rats were also assessed in PM-DAT at 6 weeks after SEVO exposure. We found that post SEVO exposure, neonatal rats that had received AAV-as-miR-19-3p exhibited significantly better performance in time spent in aversive arm at training period (Figure 6C), and in time spent in open arm at testing period (Figure 6D), compared to rats that had received AAV-null. These results suggest that both learning and memory of the neonatal rats are improved by miR-19-3p depletion after SEVO exposure. BDNF levels were examined in the rat brain 6 weeks after SEVO exposure, showing increases in BNDN levels in rats that had received AAV-as-miR-19-3p than rats that had received AAV-null (Figure 6E). Ki-67 staining was performed, showing increases in proliferating neuron cells the hippocampal area of rats that had received AAV-as-miR-19-3p than those that had received AAV-null, 6 weeks after SEVO exposure, by representative images (Figure 6F), and by quantification (Figure 6G). Together, these data suggest that abolishment of miR-19-3p upregulation protects neuron proliferation, and learning and memory of rats that were exposed to SEVO.

## DISCUSSION

Accumulating evidence have particularly shown that SEVO exposure may have non-negligible effects on the developing brain in either embryonic stage [1,4,19,20] or neonatal period [2,3,5,7,21–24]. Although different mechanisms have been proposed for the early-age-exposure-to-SEVO-induced impairment of the learning and memory ability, none of the previous studies has focused on the alteration of proliferation potential of neurons.

Here, in SEVO-exposed neonatal rat brain, we detected significant attenuated neural cell proliferation, which is critical for early brain development. Decreased number of functional neural cells may lead to inferior brain function, e.g. learning and memory, as shown in the Morris water maze test and PM-DAT. Morris water maze is a behavioral assessment mostly applied to rodents. It nicely reflects the cognitive function, spatial learning and memory ability of the animals with great accuracy [25]. PM-DAT is also a great animal model of learning and memory assessment [26]. Combination of two methods faithfully evaluated the ability of learning and memory potential of the rats. In addition, BDNF, a neurotrophin that regulates development, regeneration, survival and maintenance of neurons, was used as an additional marker for neonatal brain development and function. BDNF could protect neonatal rats from 1-methyl-4-phenylpyridinium (MPP+)-induced striatal damage and oxidative stress [27]. BDNF also enhances the neurite outgrowth on immature astrocytes [28], and umbilical cord blood mesenchymal stem cells (UCB-MSCs) expressing BNDF promote the recovery of neurological function following hypoxic-ischemic brain damage [29], suggesting a role in promoting neural cell proliferation and regeneration. In the current study, decreases in BDNF levels in the brain from the rats exposed to SEVO may result from Akt/GSK-3beta signaling pathway-dependent suppression of BDNF expression [17,30], or indirectly [31].

Among all CDKs and cyclins, we specially detected downregulation of CCNA2 dose-dependently by SEVO exposure. There are two types of CCNA, in which CCNA1 is expressed during meiosis and embryogenesis while CCNA2 is expressed in dividing somatic cells. Thus, the downregulation of CCNA2 here is apparently associated with the decreases in neuron cell proliferation. CCNA2 binds to CDK2 during S phase to mediate initiation and progression of DNA synthesis, and binds to CDK1 to trigger CCNB1-CDK1 activation to induce chromatin condensation and breakdown of the nuclear envelope, which is required for G2/M phase transition to finalize a cell cycle with cell splitting. Thus, reduced CCNA2 may inhibit cell proliferation at two points, resulting in stagnation of the cells in S phase. It may be interesting to test the distribution of SEVO-exposed neural cells in the cell cycle in future studies to examine this hypothesis.

Since CCNA2 mRNA was unaffected by SEVO, it is proposed that a post-transcriptional control of CCNA2 may be present. Although protein degradation and mRNA modification may both contribute to this phenomenon, our approach to examine the involvement of a miRNA-mediated knockdown of CCNA2 showed that the latter may be responsible for the unpaired mRNA and protein levels of CCNA2. Using TargetSan, we found that rat CCNA2 had 18 targeting miRNAs. Among these 18 miRNAs, only the levels of miR-19-3p significantly went down after the exposure to SEVO. Interestingly, miR-19-3p has been shown to be associated with cell cycle regulations in many settings [32–35], but CCNA2 as a target of miR-19-3p has not been reported. Here we used AAVs to overexpress as-miR-19-3p in rat brain, since plasmids do not allow long-term transduction of the cells, while AAVs’ mediated transduction has been shown to be persistent, induce limited inflammation, and clinically safe [36]. However, although here we found positive results on miR-19-3p-depleted, SEVO-exposed rat brain, targets of miR-19-3p other than CCNA2 may also contribute to the final outcome. In the future, a systematic analysis on miR-19-3p target genes may provide a better understanding of the functional role of miR-19-3p in treating SEVO-exposed animals.

## MATERIALS AND METHODS

### Protocol approval

All the experimental methods including animal experiments have been approved by the research committee of Shandong University.

### Animals

All experiments were performed in strict accordance with the Care and Use of Laboratory Animal Guideline, issued by Shandong University. Male Sprague-Dawley rats were raised at Laboratory Animal Center of Shandong University and used at 4 weeks of age for the experiments in the current study. The animals were kept under a 12-h light-dark cycle, and 22 ± 1°C room temperature. The rats in the control group were placed in a chamber flushed continuously with air alone for 4 hours, while the rats in SEVO group were exposed in 5% SEVO and air for 4 hours. Same flow rate of 1.5L/min was applied for either group.

### Cell culture and treatment

HCN-2 is a human cortical neuron cell line purchased from ATCC (ATCC, Rockville, MD, USA). HCN-2 cells were cultured in Dulbecco’s Modified Eagle’s Medium (DMEM) suppled with 15% fetal bovine serum (Invitrogen, Shanghai, China) in a humidified chamber with 5% CO_2_ at 37 °C.

HCN-2 cells were exposed to SEVO in a gas-tight chamber placed in the incubator at 37 °C, and the concentration of SEVO was precisely manipulated via a sevoflurane-specific vaporizer (Yu Yan Instruments, Shanghai, China). The gas mixture contained 5% CO_2_, 21% O_2_, and balanced nitrogen. The concentrations were maintained throughout experiments using an infrared Ohmeda 5330 agent monitor (Coast to Coast Medical, MA, USA).

### Plasmids and viruses

Plasmids carrying miR-19-3p or null or antisense for miR-19-3p (as-miR-19-3p) were constructed using corresponding sequence. Sequence for miR-19-3p: 5’- UGUGCAAAUCCAUGCAAAACUGA-3’; Sequence for as-miR-19-3p: 5’- UCAGUUUUGCAUGGAUUUGCACA-3’. For generation of adeno-associated virus (AAV), a pAAV-CMV-GFP plasmid (Clontech, Mountain View, CA, USA) was used. The backbone also carries a GFP reporter to allow visualization of the transduced cells by green fluorescence. Human embryonic kidney 293 cell line (HEK293, ATCC) was used for virus production. The pAAV-CMV-GFP plasmid, a packaging plasmid carrying the serotype 9 rep and cap genes, and a helper plasmid (Applied Viromics, LLC. Fremont, CA, USA) were triply transfected HEK293 cells by Lipofectamine 3000 reagent (Invitrogen). A CsCl density centrifugation method was used to purify virus, the titration of which was determined by a quantitative densitometric dot-blot assay. AAVs (5X10^11^ viral particles in 100µl) were intracranially injected into the bilateral hippocampi of the rats.

### Western blot

The cells or rat brain tissue were homogenized in protein lysis buffer to obtain protein, the concentration of which was determined using a BCA protein assay kit (Bio-rad, Beijing, China). Western blot was done as routine, using primary antibodies including rabbit anti-BDNF, anti-CCNA1, anti-CCNA2, anti-CCNB1, anti-CCNE1, anti-CCNE2, anti-CDK1, anti-CDK2, anti-CDK4 and anti-α-tubulin (Cell Signaling, San Jose, CA, USA). The secondary antibody was HRP-conjugated anti-rabbit (Jackson ImmunoResearch Labs, West Grove, PA, USA). The presentative blot images were randomly selected from 5 individuals. NIH ImageJ software (Bethesda, MA, USA) was used for image acquisition and densitometric analysis of the gels.

### Ki-67 staining and quantification

The hippocampal area of rat brain, or cultured HCN-2 cells were stained with mouse anti-human Ki-67 antibody (Abcam, Shanghai, China). Quantification was done based on counting that met 2 rules, at least 5000 total cells and at least 50 positive cells.

### Quantitative PCR (RT-qPCR)

Total RNA was extracted using miRNeasy mini kit (Qiagen, Hilden, Germany), and then reversely transcribed to complementary DNA (cDNA) using miScript II RT Kit (Qiagen). Quantitative PCR was performed in duplicates with QuantiTect SYBR Green PCR Kit (Qiagen). All primers were purchased from Qiagen. Values of genes were determined by sequential normalization to α-tubulin and the experimental controls.

### Bioinformatics and dual luciferase-reporter assay

TargetSan was used to predict the miRNA binding targets, as described [37]. Luciferase-reporters including the wildtype and mutate 3’-UTR were constructed (Promega, Beijing, China), and used in a dual-luciferase reporter gene assay kit (Promega), according to the manufacturer’s instruction.

### Morris water maze test

Morris water maze test was performed according to the published standard [38]. The route and time spent in Morris water maze were recorded. Briefly, the circular pool of 200 cm in diameter and 70 cm in depth was filled with warm opaque water (25 ± 1.0°C) and divided into four quadrants. A platform of 10cm in diameter was placed at the center of the pool. The examined rats were subjected to three consecutive training days to allow them to be familiar with seeking and perching on the hidden platform that was maintained in a fixed location. At the start of each trial, rats were placed in the pool facing the wall and were allowed to swim for 60 seconds or until the platform was found. If the rat did not find the platform during the trial, it was guided to the platform and stayed there for 15 seconds. The time to reach the platform (latency) and path lengths were recorded by software.

### Plus-Maze discriminative avoidance task (PM-DAT)

A wood-made modified plus-maze was used in the plus-maze discriminative avoidance task (PM-DAT), as described [25]. In the training session, rats were placed at the center of the apparatus, and received both the illumination of the 100 W light and cold air blow when they entered the enclosed arm containing the lamp and the hair dryer. Twenty-four hours after the training, rats were placed in the same position in the same room for 3 minutes without these aversive stimuli when they entered the enclosed arm with presence of non-illuminated lamp and the hair dryer. The percentage of time spent in the aversive enclosed arm during training and testing was recorded respectively for assessment of learning and memory.

### Statistics

GraphPad Prism 6 (GraphPad Software, San Diego, CA, USA) was used for statistical analysis. Analysis was done by one-way ANOVA with a Bonferroni correction, followed by Fisher’s Exact Test upon necessity. All values are depicted as mean ± standard deviation from 5 to 10 individuals and are considered significant if p < 0.05.

### Data availability statement

All data generated or analyzed during this study are included in this published article.
